# **Health Departments and PrEP:** A Missed Opportunity for Public Health

**DOI:** 10.1017/jme.2022.39

**Published:** 2022

**Authors:** Carri Comer, Ricardo Fernández

**Keywords:** Health Department, PrEP, Access, 340B, PrEP Affordability, Public Health, HIV Prevention

## Abstract

The paper identifies common barriers and challenges to Pre-Exposure Prophylaxis (PrEP) uptake and offers considerations for state and local public health departments to address barriers and retool infrastructure to increase access to PrEP to new users. Authors identify synergistic opportunities with federal agencies and funders to advance PrEP-related HIV prevention efforts, that prioritize strategies and investments to provide PrEP to people who could benefit from the intervention but are unaware of PrEP or struggle to access it. Barriers discussed and examined include financing strategies to reduce financial burden of PrEP medication, expanding PrEP access and outreach beyond clinical settings, and increasing the network and reach of the provider community to serve people we oppress through policy choices and discourses of racial and socioeconomic inferiority.

## The Failure to Mount a Public Health Approach to PrEP Access in the U.S

Increasing access to PrEP is critical to meeting the ambitious goals of the Ending the HIV Epidemic initiative.^1^ And, yet, as of 2019, only 23% of individuals eligible for PrEP according to Centers for Disease Control and Prevention (CDC) guidelines are actually taking PrEP. At the same time, disparities across race/ethnicity, gender, social constructs, and geography are growing.^2^ The public health approaches to PrEP taken so far in most jurisdictions throughout the U.S. have been insufficiently resourced. PrEP was first approved in the U.S. in 2012, during a time of health care innovation and transformation as a result of passage of the Patient Protection and Affordable Care Act (ACA). State and local health departments were tasked with rolling out PrEP interventions while adapting to a changing and variable healthcare landscape created by the ACA. Primary care and other traditional health care systems have relied on health departments, and federally-funded programs administered by community-based organizations, to not only provide specialty care and care coordination to achieve prevention and control goals for HIV, STIs, immunizations, tuberculosis, and family planning, but also serve oppressed, disadvantaged, and racial/ethnic communities that would otherwise have been entirely unserved. The tension the ACA unearthed between the role of public health and the role of traditional health care systems — and which systems and providers are better poised to reach certain communities — played out in the PrEP space too. PrEP is a biomedical intervention that necessitates some clinical services, but the communities most in need are not necessarily connected with clinical systems of care.

The federal government recognizes the importance of PrEP in its Ending the HIV Epidemic (EHE) initiative which began in 2019. The EHE initiative emphasizes an increased use of PrEP as the key HIV prevention intervention. However, in many ways these strategies were designed to help states better cope with existing challenges to PrEP access in the healthcare system but not actually remove those barriers. Specifically, federally funded activities focus on scaling up science-based strategies and do very little to address the key institutional and societal factors of racism, stigma, education, substance use, location, and financing. The COVID-19 response has resulted in a significant boost in the capacity of infectious disease programs in state health departments. While capacity has been limited during COVID-19 surges, ultimately the increased workforce investment may position state health departments to build off of the COVID-19 response to accelerate PrEP access and uptake. To succeed, health departments must be willing to consider new practices and models for purchasing, managing, and providing other public health services like PrEP, vaccines, naloxone, STI treatment medications, and other preventative products. They must also demand that a federal response address systemic barriers to PrEP and that future federal funds are sharply focused on increasing uptake of PrEP for new users.

## Reasons People Are Not Using PrEP

There are many reasons why PrEP uptake is so low in the U.S., particularly for communities that have been marginalized and disenfranchised who often live further from traditional health care sites, experience language and cultural barriers to care, and in many cases, fear and do not trust resources and systems that claim to provide support. Systemic racism impacts every facet of the U.S. health care and public health systems and creates structural disadvantages in access to PrEP based on race.^3^ These systemic inequities impact how and where PrEP programs are set up and who can access them. These inequities are reflected in the following structural challenges that state and local public health departments should address.

The high cost of the medication is a persistent challenge to PrEP access. List prices for the brand-name oral medications — tenofovir disoproxil fumarate/ emtricitabine/ (TDF/FTC) and tenofovir alafenamide/ emtricitabine (TAF/FTC) — without health insurance, discounts, or coupons are $1,700 and $1,900 per month respectively. In December 2021, the Food and Drug Administration (FDA) approved long-acting cabotegravir, the first long-acting injectable product for PrEP.^4^ However, at a list price of $3,500 per dose and with few health insurance plans covering the treatment, the cost is a barrier to widespread access. While a generic version of tenofovir disoproxil fumarate/ emtricitabine (TDF/FTC) is available at an acquisition cost of $26 per month,^5^ it has not dramatically changed the access picture in the U.S. This could be, in part, because of a combination of financial incentives for 340B providers to prescribe brand-name products and aggressive manufacturer marketing campaigns. The 340B program is a federal program that allows qualifying entities (primarily clinical safety net providers serving low-income communities) to access steep discounts on most prescription drugs. The program can sometimes create incentives to prescribe higher cost medications because 340B entities are able to purchase the drug at a very steep discount, but seek reimbursement from payers at a usual and customary price, which is often much higher for brand-name drugs.^6^ For PrEP, there is evidence to suggest that providers continue to prescribe high-cost brand-name medications, even when lower cost generic TDF/FTC is the appropriate regimen and may be covered in full by the insurer.^7^

Pivoting entirely to the latest biomedical interventions instead of staying focused on efforts to increase initial uptake of PrEP and support needs for PrEP adherence among racial and sexual minorities may be short-sighted and contribute to stagnating progress on PrEP scale up. Instead, federal partners should explore national approaches that provide widespread access, through programs like the federal Vaccines for Children program (VFC) model. This could offer more transparency, protection for consumers, and budget controls for PrEP, allowing for eventual sustainable access to all PrEP products.

Another challenge is that federal funds have been restricted when it comes to what PrEP services can be funded by health departments, creating a patchwork system of access with many gaps in services. CDC HIV prevention funds cannot be used to purchase PrEP medications. Until recently, CDC funds could not pay for PrEP related screenings, counseling, interventions, or case management services.^8^ The Health Resources and Services Administration funds the Ryan White HIV/AIDS Program (RWHAP), which supports services for people living with an HIV diagnosis, limiting the ability of the program to fund PrEP medications, screenings, counseling, interventions, or case management services. These federal funding restrictions and the variability of what different organizations can provide results in a patchwork of different services to support PrEP. The funding restrictions have also caused an identity crisis for state and local health departments who have been on the frontlines of providing HIV prevention education, counseling, and condoms, but have been largely hamstrung when it comes to PrEP, in part, because of the funding conundrum described above.

Despite the expertise state and local health departments have in providing HIV, STI, immunizations, TB, and family planning services to communities who have experienced discrimination and exclusion from traditional systems, time and again funding initiatives for PrEP focus on primary care. While community health centers and other primary care providers are critical for PrEP access, they are simply not sufficient to reach individuals not already engaged with the healthcare system. Relying on community health centers and Ryan White HIV/AIDS Program providers may have seemed like low-hanging fruit to federal policy makers charged with launching an ambitious initiative to end new HIV infections by 2030, but a broader provider network is needed. State and local health departments may need to examine their funding to identify resources to build capacity and competencies to support PrEP uptake or build capacity within to provide PrEP services.

The inability to use federal funds for direct service provision or drug purchasing requires most states to depend on primary care and other health care systems to prioritize PrEP, exacerbating yet another major challenge when it comes to PrEP access. The nation’s PrEP response has relied too heavily on primary care providers and community health centers. This is concerning since primary care and traditional health systems are well known hot spots for discrimination and stigma for racial and sexual minority communities.^9^ Despite the expertise state and local health departments have in providing HIV, STI, immunizations, TB, and family planning services to communities who have experienced discrimination and exclusion from traditional systems, time and again funding initiatives for PrEP focus on primary care. While community health centers and other primary care providers are critical for PrEP access, they are simply not sufficient to reach individuals not already engaged with the healthcare system. Relying on community health centers and Ryan White HIV/AIDS Program providers may have seemed like low-hanging fruit to federal policy makers charged with launching an ambitious initiative to end new HIV infections by 2030, but a broader provider network is needed. State and local health departments may need to examine their funding to identify resources to build capacity and competencies to support PrEP uptake or build capacity within to provide PrEP services.

## What Should the Federal Government and State and Local Public Health Departments Do to Reverse Course on PrEP?

The proposal from Killelea and colleagues for expanding access to PrEP prioritizes population health approaches to increase access to PrEP.^10^ Universal purchasing and distribution strategies offer opportunities to stock and deliver PrEP medications in key settings outside of traditional health care delivery system walls. State and local public health department HIV programs could adopt the VFC framework and financing model could guide universal coverage and purchasing agreements between state and local public health departments, Medicaid programs, and insurers to assure that PrEP is available to anyone that wants it.

The U.S. must shift its PrEP access strategy from overreliance on primary care providers. Traditional health care systems have fundamentally relied on health departments to perform core public health activities and serve communities who have experienced discrimination and exclusion from traditional systems, such as racialized people and people of gender or sexual minority. Expecting the health care delivery structure to comfortably and swiftly merge into the public health activities lane would be a mistake. State and local public health departments should instead consider more balanced strategies that also infuse resources into organizations that already interact, engage, and serve communities that historically relied or currently rely on health departments and other delivery methods outside the health care system for health care, including mobile outreach units, opioid treatment programs, university student clinics, and STD clinics.

A new approach to PrEP must fund organizations that are led by individuals most impacted by HIV or serve those communities wherever possible. Like the VFC program, a federal purchasing and delivery system for PrEP could support increased access to PrEP for *key populations*, specifically Latinx/Hispanic Americans, Black Americans, trans, nonbinary, and genderqueer people, people who actively use drugs, people who are unhoused, people who engage in transactional sex, people living in rural regions, and people living with lower income. Establishing contractual relationships with entities that these communities already interact with and trust could be a challenge for many state and local public health departments, and funding for technical support will likely be needed. The federal government could facilitate these contractual relationships by removing restrictions that prevent subawards or cap the amount of subawards and support more flexible funding partnerships with community-based organizations serving minority or high-risk populations. The status quo will perpetuate existing systems of oppression and lead to further widening of disparities. White state and local public health program managers and decision-makers must actively avoid business as usual PrEP programming that relies on standard systems and, in doing so, benefits White individuals at the expense of focus and attention on communities disproportionately impacted by HIV.

As part of a commitment to meet people where they are and in keeping with recent updates to federal PrEP guidelines to streamline clinical prescribing practices for PrEP,^11^ state and local public health departments should consider the opportunities to support low-threshold access to PrEP, such as statewide standing orders for PrEP and law and regulation changes to allow PrEP to be dispensed without a prescription. Prioritizing non-clinical one-stop-PrEP options will be necessary to increase uptake. The federal government could offer guidance to support distribution of PrEP medication in non-clinical settings and without on-site supervision of a licensed practitioner instead of requiring states to do this independently.

The only way to support non-clinical providers to expand access to PrEP is to ensure they are adequately funded. As discussed above, the 340B program has provided a reimbursement stream for the providers that qualify, but it has also forced dependence on high-cost medications and leaves out a large swath of providers who are not eligible for the program. Many persons from historically excluded communities, particularly racial and sexual minorities, and uninsured or underinsured persons, access care through public health clinics such as family planning and sexual health/STD clinics. The federal government will likely need to provide additional funding to support training, language access tools and resources, workflow updates, and additional reporting requirements. Funders should also consider providing additional funding and/or leveraging existing funds specifically designated to establishing more sexual health/STD clinics across the country.

Finally, the federal government and state and local public health departments must center the needs of key populations in any effort to scale up PrEP access. Many state and local public health departments fund activities that obtain input from their communities. The input received often does not include voices of those least likely to be seeking services and those with the greatest needs. State and local public health departments that are serious about leveraging PrEP to end the HIV epidemic must find ways to seek out and learn from the voices of people who could benefit from PrEP, but are not accessing it.

## Conclusion

Medications that prevent HIV acquisition have been available for nearly 10 years. Like most resources that improve health and quality of life in the U.S., there is not equitable access for all communities. PrEP should be broadly available through a network of access points able to meet people where they are to effectively reach underserved communities.

State and local public health departments have a great deal of influence to lead conversations, ensure communities are heard and represented, and that services needed are available. While there is variability in different jurisdictions, health departments have some power to effectuate change in their communities to increase utilization of PrEP. It is crucial that health departments do what they can to increase the uptake of PrEP. A new purchasing and distribution model could absorb financial and administrative burden so that key populations can be served and meaningful work can be done within communities and health systems to achieve the goals of ending the HIV epidemic.

## Note

The authors do not have any conflicts of interest to disclose.
